# The Effect of the Coronavirus 2019 (COVID-19) Pandemic on the Number and Characteristics of Orthopedic Trauma Patients in a Tertiary Care Hospital in Istanbul

**DOI:** 10.7759/cureus.12569

**Published:** 2021-01-08

**Authors:** Engin Carkci, Barış Polat, Ayşe Polat, Barış Peker, Yusuf Öztürkmen

**Affiliations:** 1 Orthopaedics and Traumatology, Istanbul Training and Resarch Hospital, Istanbul, TUR; 2 Orthopaedics and Traumatology, Dr. Burhan Nalbantoglu State Hospital, Nicosia, CYP; 3 Orthopaedics and Traumatology, Dr. Akçiçek State Hospital, Kyrenia, CYP; 4 Orthopaedics and Traumatology, Istanbul Training and Research Hospital, Istanbul, TUR

**Keywords:** covid-19, turkey, trauma, fracture

## Abstract

Objective

The aim of this study was to investigate the distribution of the causes of traumatic fractures, types of fractures, and fractures requiring surgery occurred during the one month period following the measures taken by the government due to the COVID-19 pandemic and to compare these data with that of the previous year.

Patients and methods

The number of patients with fractures, the distribution of these fractures and the number of patients who had surgical indications and underwent a surgery were identified. Patients’ age, gender and the type of trauma they were exposed to were determined.

Results

While there were 117 patients who were diagnosed with a fracture during the one month period in 2020, 619 patients had presented to our department during the same period in 2019. When compared to 2019, the prevalence of the trauma mechanisms for falling from height, traffic accident, low-energy trauma, firearm injury and sports injuries decreased by 91.7%, 85.7%, 80.3%, 80.0% and 72.7%, respectively, in 2020. A statistically significant increase was proportionally observed in pediatric supracondylar humerus, boxer’s and hip fractures (p<0.001).

Conclusion

Compared to the same period last year, although the number of patients with fractures decreased significantly following the implementation of most of the public quarantine measures, a statistically significant increase was observed in pediatric supracondylar humerus, boxer and hip fractures on a proportional basis.

## Introduction

The new coronavirus disease (COVID-19) was diagnosed in Wuhan, China in December 2019 after the development of unknown pneumonia cases [1]. It's worldwide incidence has increased rapidly due to its high rate of spread and pathogenicity. Finally, on March 11, 2020, the World Health Organization reported that 118,000 cases were detected in 114 countries due to COVID-19 and declared the disease a pandemic.

The COVID-19 pandemic has had enormous effects not only in the field of health, but also in terms of social, economic and social psychology. As is the case in the whole world, the Turkish government took a series of measures to control the pandemic. It ordered people to stay home and restricted human movements except for work, urgent needs and health reasons. Many hospitals have been declared pandemic hospitals. A large part of most hospitals was reserved solely to serve coronavirus patients. Elective surgeries were postponed throughout the country, and other patients were treated only in emergencies. In sequence, schools were closed, collective events organized for religious, sporting or other purposes were canceled, people over the age of 65 and under 20 were prohibited from going out to the streets. As of April 11, curfew has been declared on weekends and public holidays [2]. In Turkey, presentations to the emergency room are usually due to traffic accidents and sports injuries [3,4]. As a result of the new arrangements in social life, the number and distribution of patients with fractures admitted to the emergency department due to trauma inevitably changed. According to our hypothesis, with the effect of all measures, there has been a numerical decrease in all fractures, but when evaluated on a proportional basis, some fractures may have increased. On this basis, this study aimed to investigate the distribution of the causes of traumatic fractures, types of fractures, and fractures requiring surgery for patients who presented due to orthopedic trauma and were diagnosed to have a fracture in our hospital, during the one month period following the weekends and public holidays curfew measures taken by the government on April 11 due to the COVID-19 pandemic and to compare these data to that of the previous year.

## Materials and methods

Study design and patient selection

After getting the approval from the Ethics Committee of Istanbul Training and Research Hospital (No:2418), patients who presented to our emergency department as a result of orthopedic trauma during the one month period (from April 12 to May 12) of curfew and the patients from the same period of the previous year were examined retrospectively. The study was conducted in accordance with the principles of the Declaration of Helsinki. Based on the physical examinations and radiographs, the number of patients with fractures, the distribution of these fractures, and the number of patients who had surgical indications and underwent surgery were determined in addition to patients’ age, gender and the type of trauma they were exposed to. Trauma mechanisms were classified as traffic accident, sports injuries, low-energy trauma, gunshot injuries, and fall from height. Falling from a height of 1 meter or less than 1 meter was considered as low-energy trauma, while falling from a height of more than 1 meter classified as falling from a height. Fractures detected within the curfew period were compared to those from the same period last year. All patient data was retrieved from the hospital’s medical archives, the initial evaluation of the patients at the trauma center was made by an emergency specialist, and the diagnosis and treatment by at least one orthopedic specialist. Fracture diagnosis was made based on physical examination and x-ray results.

Statistical analysis

Descriptive statistics were calculated on the demographics, trauma mechanism and fracture types in patients who presented to the orthopedics and traumatology department in the same period of 2019 and 2020. The Shapiro-Wilk test was used to assess the distribution of age variables. Since the variables did not show a normal distribution, the Mann-Whitney U test was applied to compare these variables statistically. Pearson’s chi-square test and Fisher’s exact test (for expected cell counts less than 5) were used to compare the prevalences of the trauma mechanism and fracture types according to the number of applicants in any period. The alpha level was set at 0.05, and statistical analysis was done using the SPSS (Statistical Package for the Social Sciences, IBM) v.18.0 software.

## Results

While there were 117 patients diagnosed with a fracture during the one month period in 2020, 619 patients had presented to our department during the same period in 2019, which means an 81.1% decrease. The average age of the patients admitted to the emergency room was 39.55±27.14 years in 2020, while it was 37.96±23.40 years in 2019. When compared to 2019, the prevalence of the trauma mechanisms for falling from height, traffic accident, low-energy trauma, firearm injury and sports injuries decreased by 91.7%, 85.7%, 80.3%, 80.0% and 72.7%, respectively, in 2020 (Table 1). The number of patients diagnosed with a fracture who underwent surgical treatment was 22 in 2020 and 108 in 2019, which showed a 79.6% decrease. The distribution of age, gender and trauma mechanisms of the patients who required a surgical treatment is summarized in Table 2.

**Table 1:** Distribution and comparison of the age, gender and trauma mechanism in patients who had a traumatic fracture during the one-month periods in 2019 and 2020 that were investigated.

		Patients with a fracture						
		2019			2020			Percentage change
		N	%	Mean±SD	n	%	Mean±SD	
Age				37.96±23.40			39.55±27.14	
Gender	Female	262	42.3		47	40.2		-82.1%
	Male	357	57.7		70	59.8		-80.4%
	Total	619	100.0		117	100.0		-81.1%
Trauma mechanism	Traffic accident	70	11.3		10	8.5		-85.7%
	Sports injuries	22	3.6		6	5.1		-72.7%
	Fall from high	24	3.9		2	1.7		-91.7%
	Low-energy trauma	498	80.5		98	83.8		-80.3%
	Gunshot injuries	5	0.8		1	0.9		-80.0%
	Total	619	100.0		117	100.0		-81.1%

**Table 2:** Distribution and comparison of the age, gender and trauma mechanism in patients who required surgical intervention during the one-month periods in 2019 and 2020 that were investigated.

		Patients who required surgical intervention						
		2019			2020			2020
		n	%	Mean±SD	n	%	Mean±SD	
Age				44.06±25.06			36.91±27.86	
Gender	Female	37	34.3		9	40.9		-75.7%
	Male	71	65.7		13	59.1		-81.7%
	Total	108	100.0		22	100.0		-79.6%
Trauma mechanism	Traffic accident	27	25.0		6	27.3		-77.8%
	Sports injuries	6	5.6		1	4.5		-83.3%
	Fall from high	13	12.0		1	4.5		-92.3%
	Low-energy trauma	59	54.6		13	59.1		-78.0%
	Gunshot injuries	3	2.8		1	4.5		-66.7%
	Total	108	100.0		22	100.0		-79.6%

In terms of quantity, only the number of boxer’s fractures showed an increase. In terms of percentage, elbow, pediatric supracondylar humerus, boxer’s, tibia, hip and pelvis fractures showed an increase, with the increases in pediatric supracondylar humerus, boxer’s and hip fractures being significant (p<0.001) (Table 3).

**Table 3:** Distribution of the fracture types observed during the one-month periods in 2019 and 2020 that were investigated. *Fisher’s exact test †Pearson’s chi-squared test (contingency) PCN: percentage change in number of patients, PCR: percentage change in rate. Values that indicate an increase and significant p values are highlighted by #.

Fracture	2019				2020				Fracture (2020 vs 2019)			Fracture + surgery (2020 vs 2019)		
type	Fracture		Fracture + surgery		Fracture		Fracture + surgery		PCN	PCR	P	PCN	PCR	P
	n	%	n	%	n	%	n	%						
Clavicle	13	2.1	2	1.9	2	1.7	0	0.0	-84.6%	-0.4%	0.282^*^	-100%	-1.9%	0.689^*^
Humerus	41	6.6	5	4.6	5	4.3	0	0.0	-87.8%	-2.3%	0.336^†^	-100%	-4.6%	0.389^*^
Elbow	18	2.9	6	5.6	4	3.4	2	9.1	-77.8%	0.5%	0.210^*^	-66.7%	3.5%	0.272^*^
Pediatric supracondylar humerus	17	2.7	6	5.6	13	11.1	6	27.3	-23.5%	8.4%	0.001^*^	0.0%	21.7%	0.005^*#^
Forearm	23	3.7	9	8.3	2	1.7	0	0.0	-91.3%	-2.0%	0.140^*^	-100%	-8.3%	0.178^*^
Distal radius	104	16.8	7	6.5	10	8.5	0	0.0	-90.4%	-8.3%	0.204^†^	-100%	-6.5%	0.264^*^
Scaphoid	6	1.0	0	0.0	1	0.9	0	0.0	-83.3%	-0.1%	0.380^*^		0.0%	
Boxer’s	27	4.4	0	0.0	33	28.2	0	0.0	18.2%	23.8%	0.001^†^		0.0%	
Other fracture of the hand	52	8.4	1	0.9	0	0.0	0	0.0	-100%	-8.4%	0.001^†^	-100%	-0.9%	0.831^*^
Metacarpals	7	1.1	3	2.8	0	0.0	0	0.0	-100%	-1.1%	0.296^*^	-100%	-2.8%	0.571^*^
Other fracture of the foot	50	8.1	0	0.0	0	0.0	0	0.0	-100%	-8.1%	0.110^†^		0.0%	
Metatarsals	46	7.4	1	0.9	1	0.9	0	0.0	-97.8%	-6.5%	0.080^†^	-100%	-0.9%	0.831^*^
Calcaneus	8	1.3	2	1.9	1	0.9	0	0.0	-87.5%	-0.4%	0.359^*^	-100%	-1.9%	0.689^*^
Ankle	55	8.9	8	7.4	6	5.1	1	4.5	-89.1%	-3.8%	0.176^†^	-87.5%	-2.9%	0.352^*^
Tibia	40	6.6	16	14.8	8	6.8	2	9.1	-80.0%	0.2%	0.662^†^	-87.5%	-5.7%	0.310^*^
Patella	12	1.9	5	4.6	2	1.7	1	4.5	-83.3%	-0.2%	0.290^*^	-80.0%	-0.1%	0.411^*^
Femur	18	2.9	8	7.4	3	2.6	2	9.1	-83.3%	-0.3%	0.240^*^	-75.0%	1.7%	0.305^*^
Hip	36	5.8	25	23.1	21	17.9	7	31.8	-41.7%	12.1%	0.001^†^	-72.0%	8.7%	0.390^†^
Pelvis	15	2.4	0	0.0	3	2.6	0	0.0	-80.0%	0.2%	0.247^*^		0.0%	
Vertebra	17	2.7	4	3.7	2	1.7	1	4.5	-88.2%	-1.0%	0.229^*^	-75.0%	0.8%	0.412^*^
Coccyx	14	2.3	0	0.0	0	0.0	0	0.0	-100%	-2.3%	0.087^*^		0.0%	
Total	619	100%	108	100%	117	100%	22	100%						

## Discussion

The most important findings in this study is that after the implementation of most of the public quarantine measures, a significant decrease in the number of fractures developed after orthopedic trauma and in the number of fractures that required surgical treatment was detected (81.1% and 79.6%, respectively). Additionally, a statistically significant increase was proportionally observed in pediatric supracondylar humerus, boxer’s and hip fractures. In the literature, it has been shown that public quarantine implemented during a pandemic period also leads to a significant decrease in ocular, general and orthopaedics trauma rates [5-7]. A significant reduction was also observed in outdoor injuries such as traffic accidents and sports injuries, which occupy a large proportion in the fracture etiology. In our study, the effects of stay at home strategy for a large part of our society, excluding public officials such as health and security workers, on injuries has been emphasized.

Low-energy traumas, the biggest etiological cause in both examined periods occurred under a broad spectrum of occasions, including falling while walking, falling off the stairs, falling off the sidewalk, punching the wall or falling in the bathroom. In 2020, most of the low-energy traumas recorded as etiological causes were observed to be indoor injuries such as falling in the bathroom, off the stairs or inside the house.

During the pandemic period, all departments converted to serve as emergency services only. Elective surgeries and treatments were postponed [8]. The main purpose of this strategy was to avoid unnecessary possible virus exposure from healthcare personnel and equipment and to reserve the equipment and resources required for the treatment of COVID-19 patients. After the number of patients associated with COVID-19 started declining, studies that suggested initiating elective surgeries with a series of measures have been published [9].

Despite the public quarantine and calls for stay at home, trauma patients did not stop presenting to hospitals [10]. In terms of percentage, we observed a statistically significant increase in pediatric supracondylar humerus, boxer’s and hip fractures. Failure to comply with calls to stay at home and increased pandemic stress may have increased the tendency to violence in some parts of the society. In addition, the quarantine of the society led to the emergence of many psychological problems such as panic disorder, anxiety and depression [11]. It has been shown that psychological problems such as anxiety and depression are statistically significantly higher in patients with boxer's fracture compared to the healthy control and other fractures group [12]. The increase in the incidence of boxer’s fracture may be due to all this. Similarly, it has been shown that psychological problems such as hyperactivity disorder, anxiety and depression are significantly higher among children with a fractured limb [13]. We believe that the negative effects of the public quarantine on children’s psychology and difficulties in keeping them at home caused the increase in the number of domestic accidents and pediatric supracondylar humerus fractures. We also believe that the number of hip fractures also increased since these fractures in the elderly usually develop due to falls at home. Restriction of physical activity in the elderly leads to progressive loss of muscle mass and function. This condition is associated with osteoporosis and increased hip fracture risk [14,15].

We are of the opinion that some patients preferred not to come to the hospital due to the risk of coronavirus exposure, even after having experienced a severe trauma or life-threatening diseases such as heart attacks [16]. This may have reduced the number of hospital admissions despite having experienced a trauma.

Our study had some limitations. Our study included patients diagnosed with fractures only after clinical and radiological examinations and required surgical treatment, instead of all trauma patients. Isolated dislocations and soft tissue traumas were excluded. Although our hospital is a Level 1 trauma center in a metropolitan area, it still fails to address all injured patients. Thus, more meticulous, multi-center statistical analyses are required. However, we believe that our study is a good one that shows the effect of public quarantine on the number and distribution of orthopedic fracture patients and fracture patients that require surgical treatment.

## Conclusions

When compared to the same period last year, and after the implementation of most of the public quarantine measures, significant decreases were observed in the number of patients who were admitted to the emergency department and diagnosed with a fracture and who required surgical treatment (81.1% and 79.6%, respectively). Furthermore, a statistically significant increase was proportionally observed in pediatric supracondylar humerus, boxer’s and hip fractures. In our study, the effects of stay at home strategy for a large part of our society on injuries have been emphasized.
